# An insight into the Nomogram of Percutaneous Nephrolithotomy

**DOI:** 10.1590/S1677-5538.IBJU.2023.0398

**Published:** 2024-02-07

**Authors:** Amirhossein Shahabi, Shahab Aali

**Affiliations:** 1 Guilan University of Medical Sciences Razi Hospital Urology Research Center Rasht Iran Department of Urology, School of Medicine, Urology Research Center, Razi Hospital, Guilan University of Medical Sciences, Rasht, Iran

## To the editor

A study by Xie et al. (1). presented an excellent investigation of the practical nomogram design and the implementation of mini percutaneous nephrolithotomy (PCNL) and laser techniques to reduce complications and improve stone clearance. We want to raise a few additional considerations regarding the methodology and potential variables that could influence the study outcomes. Firstly, while the study reported the use of nephrostomy, no explanation was provided regarding implementing a double-J stent or ureteral stent. These stents may induce passive dilatation and facilitate stone expulsion during the follow-up period. It would be worthwhile to explore the effect of double-J and ureteral stents on stone clearance rates and assess their potential impact on the results (2). Considering these variables might provide further insights into optimizing patient outcomes.

Secondly, in the group of patients who had previously undergone surgery, careful attention should be given to analyzing the characteristics of the prior stones in terms of their hardness. The degree of stone hardness can significantly influence the effectiveness of PCNL and contribute to variable outcomes (3). By incorporating the analysis of stone hardness, future studies can better understand the relationship between stone characteristics and treatment success rates, enabling the development of more tailored treatment strategies. Additionally, the positioning of patients, both prone and supine, could potentially have an impact on treatment outcomes (4). Exploring the effect of a patient's position as one of the variables could provide valuable insights into the optimal positioning for achieving successful stone clearance in future studies.

In conclusion, we appreciate the valuable contributions made by Xie et al. on developing a nomogram for predicting the risk of adverse outcomes in patients with residual stones following PCNL. However, we suggest further investigations consider the impact of double-J and ureteral stents on stone expulsion rates, analyze the degree of stone hardness in patients with prior surgeries, and explore the effect of the patient's position on treatment outcomes. By addressing these considerations, we will enhance the accuracy and applicability of predictive models and contribute to advancing personalized treatment approaches.

The Author
